# The beneficial effects of square dance on musculoskeletal system in early postmenopausal Chinese women: a cross-sectional study

**DOI:** 10.1186/s12905-022-01832-9

**Published:** 2022-06-21

**Authors:** Jie Sun, Chen Yao, Ziping Wang, Jiancheng Wu, Bo Zhang, Zhenyu Zhou, Fan Liu, Yafeng Zhang

**Affiliations:** 1 Medical School of Nantong University, Nantong City, Jiangsu Province People’s Republic of China; 2 Department of Orthopaedics, The Affiliated Hospital of Nantong University, Nantong City, Jiangsu Province People’s Republic of China

**Keywords:** Postmenopausal women, Square dance, BMD, Musculoskeletal system

## Abstract

**Background:**

This study was set to investigate the correlation between square dance and musculoskeletal system of early postmenopausal Chinese women.

**Methods:**

Chinese postmenopausal women, who had been without menstruation for 1–10 years from the onset of menopause were recruited from community centers for this study. A standardized structured face-to-face interview was performed to collect demographic information, life styles, personal medical history, diet and menstrual status. Subjects who had been practicing regular square dance without participated in other sports activities for more than 2 years and over 4 h per week (usually more than 45 min per time and more than 5 times per week) were assigned to square dance group. Those postmenopausal women who had not participated in regular exercises (no more than 0.5 h per week) were recruited as the sedentary control group. Bone mineral density (BMD) of spine, total hip and femoral neck was measured by using dual-energy X-ray absorptiometry. Lower limb muscle strength was measured for the non-dominant leg, body flexibility was measured by a simple trunk bend-and-reach test, and body balance was evaluated using a single-stance test for the non-dominant leg. Independent two-tailed Student’s t-test was used for data analysis.

**Results:**

152 subjects from community centers were selected for this study and divided into square dance group (n = 74) and control group (n = 78). The square dance subjects had higher lumbar spine BMD (*p* = 0.01) and total hip BMD (*p* = 0.02) than control subjects, but there was no significant difference of femoral neck BMD (*p* = 0.48) between these two groups. Functional testing indicated that square dance subjects had higher lower limb muscle strength (*p* < 0.01) and longer single-stance time (*p* = 0.02) than the control subjects, but there was no significant difference in trunk bend-and-reach (*p* = 0.12) between these two groups.

**Conclusion:**

Our results show that postmenopausal Chinese women can get beneficial effects, like higher BMD, stronger lower limb muscle and improved body balance ability on musculoskeletal system by participating in square dance regularly.

## Introduction

Aging of population has become an extremely tough challenge that the whole world must face in the twenty-first century. On mainland China, the residents who are 60 years old and above account for over 13% of the total population according to the latest national population census report. The age-related skeletal problems, such as osteoporosis and osteoarthritis will become more and more common in China. The structure and function of human locomotor system deteriorate with advancing age, such as bone loss, obesity reduction of muscle mass or strength and decreased coordination or balance ability, which may cause impaired physical function, higher risk of fall or fractures, reduced life quality and even higher mobility [1–3].All these factors that are more predominant in the female population after the onset of menopause. In women, almost 20% of the overall bone loss can happen and accelerate after the onset of menopause and up to half of them suffer fragility fractures [4, 5]. It is not clear whether these changes are just an effect of estrogen deficiency, or whether it is associated with malnutrition, vascular changes, or deterioration of neuromuscular function. Whatever the etiology, many studies have been performed to investigate prevention strategies including drugs, lifestyle adjustment or physical exercises [6–8].

More and more studies have reported physical exercises have multiple benefits on the locomotor system in postmenopausal women, including the prevention of muscle weakness and bone loss, thereby reducing fracture risks. The type of exercises included aerobic, stretching, resistance and balance. Different exercises focus on different effects and some exercises are span multiple categories like Yoga, Pilates and Tai Chi Chun (TCC) [9, 10]. Square dance, also known as Dama square dance, is a unique phenomenon in China and is gaining popularity with middle-aged or older women due to increased awareness of health and exercise [11, 12]. Every morning and evening, Chinese aunties, also called Dama in Mandarin, gather on open areas such as playgrounds, parks, streets or courts and dance according to loud music for hours. Every Chinese people or foreigners who went to China may see them. Young people always hate them because of the noisy music. However, elder women delight in it and seem never get tired, which is so interesting.

Square dance is classified as a moderate intensity exercise with all sorts of styles which can betangos, waltzes, line-dance, rock n' roll style jigging or just free-style bumping and grinding. In a sense, square dance is a compound exercise like TCC, while it’s relatively simple and low-impact, with a shorter learning curve. Maybe these explain why square dance is becoming more popular than TCC with over 100 million participants in China [13]. Published studies have reported that TCC was associated with reduced bone loss, lower fracture risks, improved cardiopulmonary function and better mental health in early postmenopausal women or other aged populations [14–16]. However, the similar research about square dance is rare and limited [17, 18]. Therefore, we eager to know whether or not early postmenopausal Chinese women can benefit from regular square dance, in terms of musculoskeletal system. The study performed a horizontal comparison to explore the effects of square dance on bone mineral density (BMD), muscle strength, body flexibility and balance ability in early postmenopausal Chinese women.

## Methods

### Subjects and study design

A cross-sectional study was conducted to compare the postmenopausal women who participant in square dance regularly and those who did not have this daily fitness in terms of musculoskeletal system. Chinese postmenopausal women, aged between 50 and 60 years, who had been without menstruation for 1–10 years from the onset of menopause were recruited from community centers in Nantong city, Jiangsu province, China. All subjects agreed to take part in the study and signed the informed consent before the measurement. The study protocol was approved by the Clinical Research Ethics Committee of the Affiliated Hospital of Nantong University.

A standardized structured face-to-face interview was performed to collect demographic information, life styles, personal medical history, diet and menstrual status. Then we used the China Food Composition (book 1, 2nd edition) data to calculate the calcium intake from each food category. A short version of the Health Habits and History Questionnaire (HHHQ) was used during this procedure [19]. Subjects who had been practicing regular square dance without participated in other sports activities for more than 2 years and over 4 h per week (usually more than 45 min per time and more than 5 times per week) were assigned to square dance group. Those postmenopausal women who had not participated in regular exercises (no more than 0.5 h per week) were recruited as the sedentary control group. Exclusive criteria were as the follows: subjects who had injuries affecting the body function tests, subjects who were practicing other sports activities besides square dance, subjects who were receiving hormone replacement therapy, calcium supplementation or drug treatment affecting bone metabolism, subjects who had some conditions such as hypo- or hyperparathyroidism, hypo- or hyperthyroidism, renal or liver disease.

### Outcome evaluations

#### Bone mineral density (BMD)

Standard BMD measurements were performed by using dual-energy X-ray absorptiometry (DXA) with Hologic QDR 4500 bone densitometers (Hologic, Inc. Waltham, MA). The proximal femur and lumbar spine vertebrate were evaluated. Calibration was performed daily on a lumbar spine phantom, and the coefficient of variation was 0.7%.

#### Lower limb muscle strength, body flexibility and balance ability

Lower limb muscle strength was measured for the non-dominant leg with Biodex System 3Pro isokinetic muscle strength testing instrument produced by USA. The measurement method was the same as previously reported [20]. Measurements were repeated three times and the average value was used for evaluation. Body flexibility was measured by a simple trunk bend-and-reach test. The subjects stand on the measuring board with no gap between the feet, keeping the knees straight. The lowest level their fingers could reach after trunk bending forwards and downwards was recorded. Measurements were repeated three times and the average value was used for data analysis. Body balance was evaluated using a single-stance test for the non-dominant leg. Measurement was repeated three times with a time interval of 30 s between each measurement. The average time was used for evaluation.

### Statistics

Data was presented as mean ± SD. Independent two-tailed Student’s t-test was used for data analysis. *p* Value of less than 0.05 was regarded as statistically significant, all the analyses were performed with SPSS software (version 13.0, Chicago, USA).

## Results

Finally, a total of 152 postmenopausal women were evaluated in this study, with 74 subjects in the square dance group and 78 subjects in the sedentary control group. There was no significant difference about demographic characteristics, dietary calcium intake and daily activity between two groups. The details of the comparison were showed in Table 1 (*p* > 0.05).

**Table 1:** Demographic characteristics, dietary calcium intake and daily activity between the square dance and sedentary control groups

	Square dance group N = 74	Control group N = 78	*p* Value
Age (years)	56.2 ± 3.4	55.4 ± 2.8	0.33
Height (cm)	155.2 ± 4.8	156.1 ± 5.7	0.68
Weight (kg)	55.5 ± 6.3	55.2 ± 5.8	0.84
BMI (kg/m^2^)	23.0 ± 3.1	22.7 ± 3.2	0.81
Age at menopause	50.8 ± 3.5	50.3 ± 3.4	0.56
Years since menopause	5.4 ± 3.2	5.1 ± 3.5	0.88
Calcium intake (mg/day)	891.9 ± 59.1	882.4 ± 59.2	0.32
Outdoor walking (h/day)	1.5 ± 0.4	1.6 ± 0.4	0.24

The spine BMD of square dance group is 0.874 ± 0.120, the femoral neck BMD is 0.704 ± 0.098, and the total hip BMD is 0.788 ± 0.104. The spine BMD of control group is 0.812 ± 0.118, the femoral neck BMD is 0.688 ± 0.100, and the total hip BMD is 0.742 ± 0.112. Subjects in the square dance group had higher lumbar spine BMD and total hip BMD than those in the control group (*p* = 0.01 and 0.02). There was no significant difference of femoral neck BMD between two groups (*p* = 0.48). The comparison of BMD between two groups was summarized in Table 2.

**Table 2:** Comparison of BMD between square dance and sedentary control groups

	Square dance group N = 74	Control group N = 78	*p* Value
Spine BMD	0.874 ± 0.120	0.812 ± 0.118	0.01
Femoral neck BMD	0.704 ± 0.098	0.688 ± 0.100	0.48
Total hip BMD	0.788 ± 0.104	0.742 ± 0.112	0.02

The hip extension strength of square dance group is 226.5 ± 22.6 N, the knee extension strength is 202.4 ± 19.6 N, the ankle flexion strength is 124.8 ± 21.3 N and the single-stance time is 116.5 ± 40.2 s, while the hip extension strength of control group is 191.8 ± 26.1 N, the knee extension strength is 173.5 ± 23.5 N, the ankle flexion strength is 102.4 ± 20.4 N and the single-stance time is 70.8 ± 58.4 s. Subjects in the square dance group had higher strength of hip extension (*p* < 0.01), knee extension (*p* < 0.01) and ankle dorsal flexion (*p* < 0.01), Single-stance time was significantly longer in square dance group than in control group (*p* = 0.02). The trunk bend-and-reach of square dance group is − 2.4 ± 8.8 cm, and 1.8 ± 8.0 cm in control group. No significant difference was found in trunk bend-and-reach between these two groups (*p* = 0.12). The details of results of functional tests were summarized in Table 3.

**Table 3:** Comparison of functional test between square dance and sedentary control groups

	Square dance group N = 74	Control group N = 78	*p* Value
Hip extension strength (N)	226.5 ± 22.6	191.8 ± 26.1	< 0.01
Knee extension strength (N)	202.4 ± 19.6	173.5 ± 23.5	< 0.01
Ankle flexion strength (N)	124.8 ± 21.3	102.4 ± 20.4	< 0.01
Trunk bend-and-reach (cm)	− 2.4 ± 8.8	1.8 ± 8.0	0.12
Single-stance time (s)	116.5 ± 40.2	70.8 ± 58.4	0.02

## Discussion

The results of this study showed that subjects who participated in regular square dance had higher lumbar spine BMD and total hip BMD than sedentary control subjects. In addition, the function tests indicated higher strength of lower limb muscles and better balance ability in square dance group. These results suggested that regular square dance may be beneficial for the locomotor system in early postmenopausal women.

Different from western countries, those high-impact or difficult exercises, such as running, jumping, swimming or weight lifting, may not be preferred by postmenopausal Chinese women. They prefer simple, low-impact and free-style exercises rather than go to the fitness center. As a unique Chinese exercise, TCC has been recommended to elder Chinese subjects for hundreds of years. TCC may be classified as a multicomponent exercise involving low velocity of muscle contraction and neuromuscular coordination. Square dance is something like TCC and has been becoming more popular all over China than TCC. It is still unclear whether square dance has similar benefits to multiple systems like TCC and our study provided some reference value in the field.

A retrospective cohort study conducted by Yu et al. [21] found that 24-week aerobic dance intervention could result in the lower the incidence of bone fracture through increasing BMD and decreasing fall risk for postmenopausal women. Young et al.’s [22] study found that simple, novel physical activity maintains proximal femur BMD, and improves muscle strength and balance in sedentary, postmenopausal Caucasian women, which is similar to our findings. Our results also showed that square dance was correlated with high BMD density of lumbar spine and total hip except femoral neck.

Some studies found lower BMD in elite ballet dancer [23, 24], which seems contrary to our results. But we think square dance is a relatively low-impact exercise with small size of joint movement and does not apply much bending force to femoral neck, which is different from those high-impact exercises such as running or jumping [25], which are common movements in ballet. Higher lower limb muscle strength and longer single-stance time were detected in subjects of square dance group, which suggested that square dance was associated with better muscle function and body balance. Improved muscle function and balance ability should be considered as important as increased BMD, because they can help prevent unexpected fall, decreasing risk of fragility fractures. On the other hand, the results showed no significant difference about body flexibility between two groups. Square dance is relatively skill-free without any special style and subjects may perform any actions they like easily on a comfortable state. They do not actively extend their muscles, ligaments or joints to a large range, which therefore have limited effects on the body flexibility.

Several limitations of our study exist and deserve mention. Firstly, we presented a pilot cross-sectional analysis with a limited sample size, which influenced the evidence level of this study. Secondly, due to the obvious diversity of the square dance, we have no accurate data about special intensity or style of every subject, increasing the heterogeneity of our study. Thirdly, all the data of square dance was collected by self-report, it may have personal bias. Lifestyle factors, different styles or intensities of square dance, and comprehensive dietary patterns containing calcium intake should be included in the finally evaluation. We have been performing a prospective randomized case–control study including more subjects to investigate the effect the square dance on musculoskeletal system in early postmenopausal Chinese women and the outcomes will be reported in the near future.

## Conclusion

In summary, this cross-sectional study showed postmenopausal Chinese women who participate in square dance regularly had higher BMD, stronger lower limb muscle and improved body balance ability. As a simple, free-style and multicomponent exercise like TCC, square dance may have a protective effect on postmenopausal deterioration of musculoskeletal system and is worth promoting. Further longitudinal research should be carried out to investigate the effect of square dance on bone health in early postmenopausal Chinese women.

## Data Availability

The datasets used and/or analysed during the current study are available from the corresponding author on reasonable request.

## Abbreviations

BMD: Bone mineral density
DXA: Dual-energy X-ray absorptiometry
TCC: Tai Chi Chun
HHHQ: Health Habits and History Questionnaire
